# Circulating inflammatory mediators and organ dysfunction after cardiovascular surgery with cardiopulmonary bypass: a prospective observational study

**DOI:** 10.1186/cc4857

**Published:** 2006-03-15

**Authors:** Hugo Tannus Furtado de Mendonça-Filho, Kelly Cristina Pereira, Mariane Fontes, Daniel Augusto de Souza Aranha Vieira, Maria Lucia A Furtado de Mendonça, Luiz Antonio de Almeida Campos, Hugo Caire Castro-Faria-Neto

**Affiliations:** 1 Núcleo de Pesquisa Translacional, Hospital Pró Cardíaco, Rua General Polidoro 192, Botafogo, Rio de Janeiro, RJ, 22280-000 Brazil; 2 Laboratório de Imunofarmacologia, Departamento de Farmacodinamica, Instituto Oswaldo Cruz, Rio de Janeiro, RJ, 21045-900 Brazil

## Abstract

**Introduction:**

Cardiovascular surgery with cardiopulmonary bypass (CPB) has improved in past decades, but inflammatory activation in this setting is still unpredictable and is associated with several postoperative complications. Perioperative levels of macrophage migration inhibitory factor (MIF) and other inflammatory mediators could be implicated in adverse outcomes in cardiac surgery.

**Methods:**

Serum levels of MIF, monocyte chemoattractant protein (MCP)-1, soluble CD40 ligand, IL-6 and IL-10 from 93 patients subjected to CPB were measured by enzyme-linked immunosorbent assay and compared with specific and global postoperative organ dysfunctions through multiple organ dysfunction score (MODS) and sequential organ failure assessment (SOFA).

**Results:**

Most of the cytokines measured had a peak of production between 3 and 6 hours after CPB, but maximum levels of MIF occurred earlier, at the cessation of CPB. Among specific organ dysfunctions, the most frequent was hematological, occurring in 82% of the patients. Circulatory impairment was observed in 73.1% of the patients, and 51% of these needed inotropics or vasopressors within the first 24 hours after surgery. The third most frequent dysfunction was pulmonary, occurring in 48.4% of the patients. Preoperative levels of MIF showed a relevant direct correlation with the intensity of global organ dysfunction measured by SOFA (ρ = 0.46, *p *< 0.001) and MODS (ρ = 0.50, *p *< 0.001) on the third day after surgery. MCP-1 production was associated with postoperative thrombocytopenia, and MIF was related to the use of a high dose of vasopressors in patients with cardiovascular impairment and also to lower values of the ratio of partial arterial oxygen tension (PaO_2_) to fraction of inspired oxygen (FiO_2_) registered in the first 24 hours after CPB.

**Conclusion:**

Despite the multifactorial nature of specific or multiple organ dysfunctions, MIF should be explored as a predicting factor of organ dysfunction, or even as a potential therapeutic target in decreasing postoperative complications.

## Introduction

Cardiovascular surgery with cardiopulmonary bypass (CPB) is known to be associated with many aggressive factors, including operative trauma, cardioplegia, ischemia-reperfusion injury and the contact of blood with bioactive surfaces, potentially related to platelet activation and inflammation [1]. Lipopolysaccharide (LPS) has been proved to circulate after cardiac surgery with CPB [2] and, despite substantial technological improvements in past decades, an acute transcriptional response of genes involved in the innate immune response occurs, which can lead to systemic inflammation and postoperative organ dysfunction [3].

Several circulating inflammatory mediators, including pro-inflammatory and anti-inflammatory cytokines [4] and chemokines [5], could be associated with postoperative outcomes.

Macrophage migration inhibitory factor (MIF) has been considered a central mediator of the innate immune response as a natural ligand of CD74 and has been implicated in cellular signaling and activation [6]. Moreover, MIF is known to regulate p53-induced apoptosis, which is one of the main mechanisms of immune activation control [7]. Constitutively expressed, MIF is stored in intracellular granules and is rapidly secreted under inflammatory stimuli, exerting powerful pro-inflammatory actions (reviewed in [8]). Previous studies have demonstrated that high MIF levels might have implications for pathological disorders in patients with systemic inflammatory response syndrome, severe sepsis, and acute respiratory distress syndrome [9-12].

The correlation of poor outcomes and high levels of MIF, particularly in patients in an injury setting, could be explained both by the pro-inflammatory and immunoregulatory properties of this cytokine, especially by modulating the expression of Toll-like receptor-4, a signal-transducing molecule of the LPS receptor complex, and the signal transduction cascade inherent in its activation [13]. In cardiac surgery, MIF levels six hours after CPB were associated with a poorer postoperative pulmonary short-course outcome [14].

Chemotactic proteins have an important role in evoking inflammatory responses. Monocyte chemoattractant protein (MCP)-1 has the property of chemoattracting mononuclear phagocytes, natural killer cells, T cells, mast cells, and basophils. Deleterious sequelae have been attributed to its expression. It has been implicated in transendothelial monocyte recruitment to sites of inflammation. Moreover, MCP-1 can induce respiratory burst activity and stimulate lysosomal enzyme release from monocytes and also contributes to tissue damage [15].

Soluble CD40 ligand (sCD40L), a member of the TNF family produced by activated platelets, upregulates the expression of inflammatory adhesion receptors and cellular activators, and circulates in response to cardiac surgery with CPB [16].

IL-6 is a cytokine with recognized pro-inflammatory and anti-inflammatory properties that acts as an indicator of intensity of inflammatory response [17]. IL-10 decreases the LPS-induced production of pro-inflammatory cytokines by macrophages as an attempt to modulate host response to injury [18], which is essential in re-establishing homeostasis after trauma.

The present study attempts to evaluate preoperative, perioperative and postoperative circulating levels of MIF and other inflammatory markers, to explore eventual associations between them and postoperative organ dysfunctions.

## Materials and methods

This prospective study was approved by the institutional review board and ethical committee for research of the Pro Cardiaco Hospital, Rio de Janeiro, Brazil, and was performed in a research laboratory associated with the surgical intensive care unit in a tertiary care cardiology hospital. Under informed consent, patients who underwent major cardiovascular thoracic operations from August 2004 to August 2005, in both elective and non-elective settings, were enrolled consecutively. We excluded patients with neoplastic or chronic inflammatory diseases, and those under immunomodulatory treatment or drugs, including steroids, aprotinin, coagulation factors, and non-steroid anti-inflammatory agents.

Anesthesia was performed in accordance with institutional standards [14]. After anticoagulation with sodium heparin, normothermic (34 to 37°C) CPB was performed with a continuous-flow membrane oxygenator (DMG-Shunt; DMG Equipamentos Médicos, Duque de Caxias, Brazil). The CPB priming solution consisted of mannitol and Ringer's solution, in a final volume of 2,000 ml. Cardioplegia was induced and maintained by way of St Thomas solutions.

Peripheral blood was harvested immediately before anesthesia induction, at the cessation of CPB, and 3, 6, 10, and 24 hours after the end of CPB. Serum and plasma were separated by centrifugation at 800 *g *for 15 minutes at 4°C and were kept frozen at -70°C until assayed. Serum levels of MIF, MCP-1, IL-6, IL-10, and sCD40L were assayed by an enzyme-linked immunosorbent assay sandwich technique (R & D Systems, Minneapolis, MN, USA) in accordance with the manufacturer's recommendations.

Clinical characteristics including demographics, past medical history, present surgical intervention, and subsequent hospital course were recorded. The preoperative risk profile of all patients was assessed with the European System for Cardiac Operative Risk Evaluation (EuroSCORE) [19].

Global and specific organ dysfunctions were assessed with the multiple organ dysfunction score (MODS) [20] and Sequential Organ Failure Assessment (SOFA) [21] scales, registered during the first 72 hours after surgery.

### Statistical analysis

For the assessment of sequential variations in circulating levels of the markers studied we used an analysis of variance for repeated measures with the Bonferroni test. Data are shown as means ± SEM when they had a normal distribution, or as median (range from first to third quartile) if the distribution was not normal. Differences between groups were analyzed with the Mann–Whitney *U *test. Correlations were assessed with the Spearman test. Values of *p *< 0.05 were considered statistically significant. Receiver operating characteristic curves were constructed to evaluate the diagnostic potential by means of the area under the curve and the diagnostic accuracy of the markers at various cutoff points. The optimal cutoff value was obtained by calculating the maximum value for the product of sensitivity and specificity.

## Results

The 93 patients included in the study were 66.2 ± 11.6 years old; 60 (64.5%) were male, and the body mass index was 25.9 ± 6.5. The median (range from first to third quartile) EuroSCORE was 4.58 (2 to 7). Twenty-nine patients (31%) were operated on under non-elective conditions, and did not differ from electively operated patients in age, duration of CPB, or perioperative blood and fluid balance. Patients who were subjected to cardiac surgery in urgent or emergency settings had a higher EuroSCORE (6.23 ± 0.75 versus 3.70 ± 0.35; *p *= 0.001) than electively operated patients. Concerning preoperative cardiopulmonary functional status, patients in New York Heart Association (NYHA) classes III and IV (*n *= 57; 61.2%) predominated over those in NYHA classes I and II (*n *= 36; 38.8%).

Patients were subjected to coronary artery bypass surgery (CABS) (*n *= 60; 64.5%), to CABS associated with intracardiac open-heart surgeries (*n *= 14; 15.1%), to valve replacement and/or repair (*n *= 16; 17.2%), or to aortic surgery (*n *= 3; 3.2%). The duration of CPB was 100 (80 to 130) minutes. Intraoperative fluid balance was 9.09 (6.31 to 11.36) ml/kg per hour, and 45 patients (48.4%) received perioperative hemotransfusion. Demographics and preoperative data are summarized in Table 1.

### Postoperative outcomes

The duration of CPB was directly associated with SOFA (*p *= 0.003, ρ = 0.319) and MODS (*p *= 0.004, ρ = 0.305) was measured during the first day after surgery.

For a better understanding of the postoperative outcomes in the first day, indicators of organ dysfunction were individually analyzed. According to MODS criteria for specific organ dysfunction, hematological dysfunction occurred in 82% of the patients. Circulatory impairment occurred in 73.1% of the studied population, and 51% needed inotropics or vasopressors within the first 24 hours after surgery. The third most frequent organ dysfunction was pulmonary (48.4%), followed by neurological disturbances (25.8%) and renal impairment (16%). Postoperative image-documented stroke occurred in three patients (3.2%). Postoperative levels of bilirubin remained near the normal range, and did not have a relevant effect on postoperative global organ dysfunction. The levels of C-reactive protein (CRP) were 4.9 (2.6 to 7.4) mg/dL on the first day and 14.7 (10.7 to 21.2) mg/dL on the third day after surgery.

The overall postoperative mortality in this series was 7.5% (seven patients), with no significant difference between electively and non-electively operated patients.

### Postoperative kinetic of inflammatory markers

**Figure 1:**
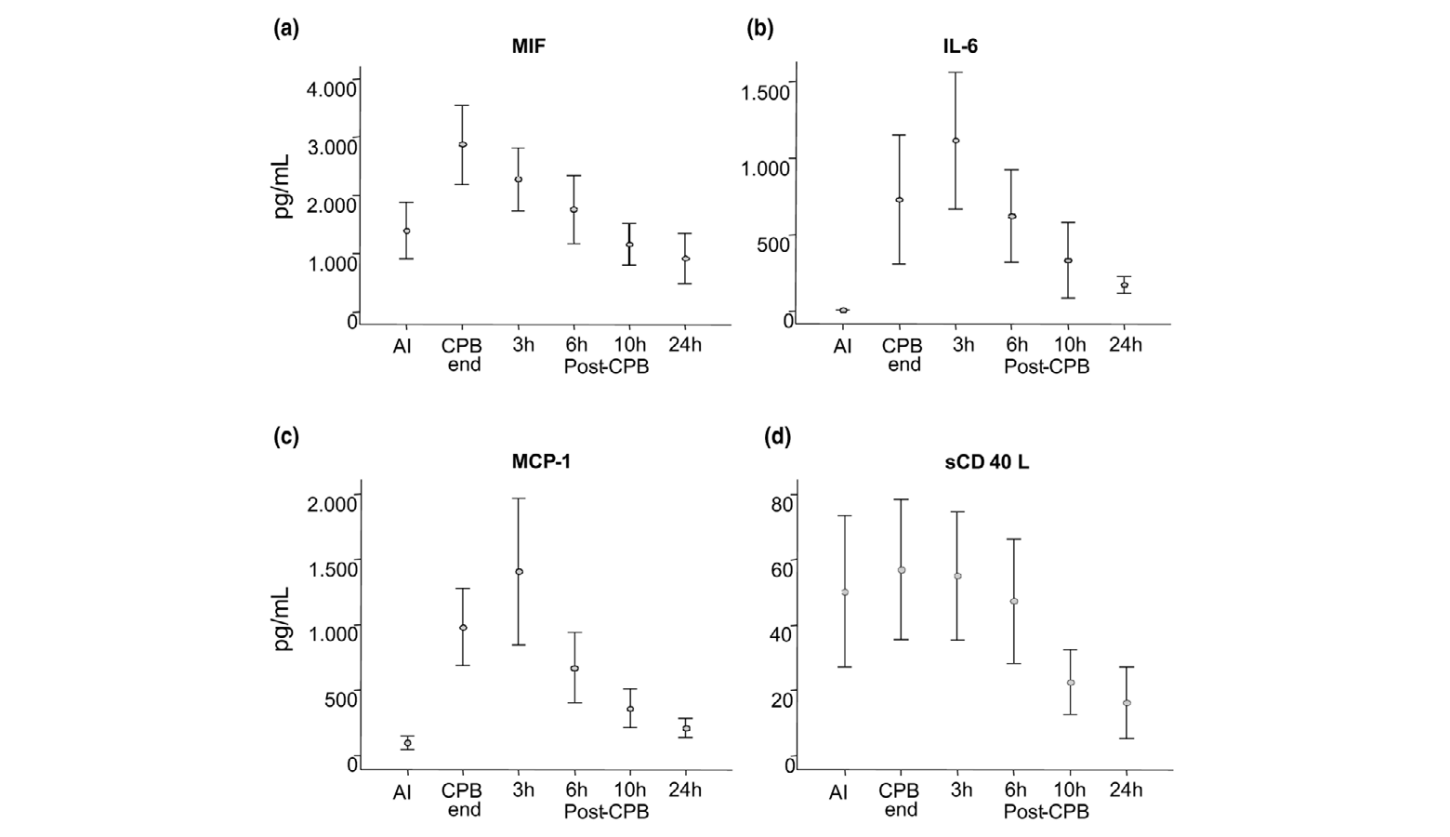
Kinetics of inflammatory mediators at anesthesia induction and after cardiopulmonary bypass. **(a) **Macrophage migration inhibitory factor; **(b) **IL-6, **(c) **monocyte chemoattractant protein-1; **(d) **soluble CD40 ligand. AI, anesthesia induction; CPB, cardiopulmonary bypass.

As shown by analysis of variance for repeated measures with the Bonferroni test (*p *< 0.05), MIF, MCP-1, and IL-6 exhibit remarkable changes related to cardiovascular surgery with CPB. Peak levels of MIF were reached at the end of CPB and were directly associated with its time course (*p *= 0.001, ρ = 0.391). Nevertheless, maximum levels of MCP-1 and IL-6 were only observed three hours after CPB. The levels of sCD40L did not change significantly until 6 hours after CPB; thereafter a decline was observed at 10 and 24 hours after CPB (Figure 1). IL-10 was detectable in very few samples and no significant change was recorded in its circulating level during the studied period.

No differences were found between inflammatory markers of elective and non-elective surgical settings, except for higher postoperative levels of IL-6 (*p *< 0.05) and for a tendency toward higher preoperative levels of MIF (*p *= 0.087) in non-elective patients.

### Preoperative levels of MIF are correlated to multiple organ dysfunctions

**Figure 2:**
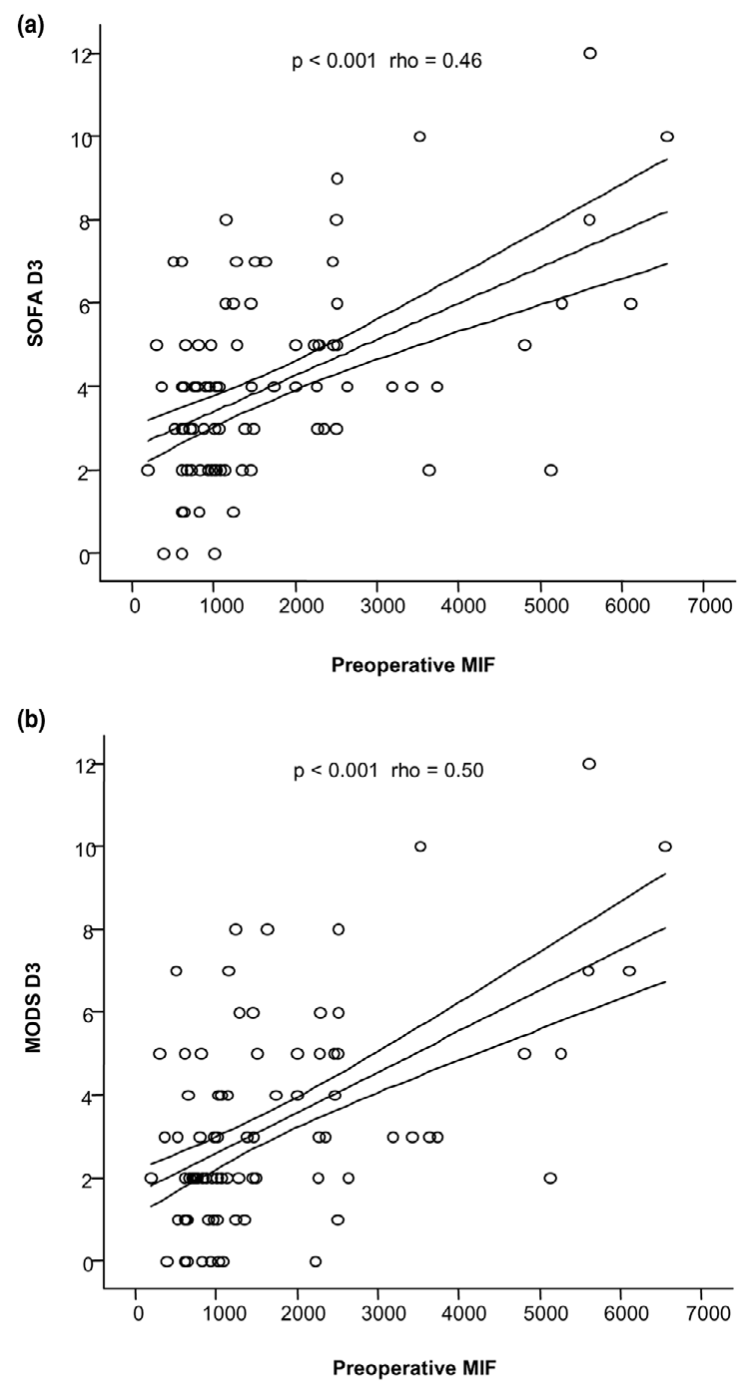
Correlation of preoperative levels of macrophage migration inhibitory factor (MIF) with postoperative organ failure scores. **(a) **Sequential Organ Failure Assessment (SOFA) at day 3 after surgery. **(b) **Multiple Organ Dysfunction Score (MODS) at day 3 after surgery. Data are shown graphically as a linear regression with 95% confidence intervals.

Preoperative circulating levels of MIF were significantly associated with the intensity of organ dysfunction, as measured by SOFA (*p *< 0.001, ρ = 0.46) and MODS (*p *< 0.001, ρ = 0.50), on the third day after surgery (Figure 2). The upper quartile of distribution consisted of patients with SOFA and MODS values higher than five. It was observed that preoperative levels of MIF were able to identify these subjects demonstrated by areas under the receiver operator characteristic curve of 0.767 ± 0.065 for SOFA and 0.794 ± 0.067 for MODS. The best cutoff value for preoperative MIF was 1,100 pg/ml (fourfold the value in healthy controls), with sensitivities of 89.5% and 93.3% and specificities of 64.9% and 66.1% for SOFA and MODS, respectively. Correspondingly, the retrospective calculations of power were 85. 3% and 97.1%.

Pressure-adjusted heart rate, the marker of circulatory dysfunction used in MODS, was not associated with circulating MIF. However, higher levels of MIF 3 hours after CPB and IL-6 10 hours after CPB were related to the need to use high doses of vasopressors within the first 24 hours after surgery (*p *= 0.017 and *p *= 0.05, respectively). Notably, levels of MIF or IL-6 were not significantly different between patients who needed dobutamine or low doses of vasopressors, and those who required no catecholamine infusions.

Analysis of the fourth quartile of distribution showed that it was composed of patients with worse postoperative outcomes. Patients who presented worse pulmonary performance (ratio of partial arterial oxygen tension (PaO_2_) to fraction of inspired oxygen (FiO_2_) less than 196) exhibited higher levels of MIF three hours after CPB (*p *< 0.001). Moreover, MIF 3 hours after CPB was inversely associated with PaO_2_/FiO_2 _ratio (*p *< 0.001, ρ = -0.383) and was directly associated with the postoperative duration of mechanical ventilation (*p *= 0.011, ρ = 0.265).

With regard to renal function, patients in the fourth quartile exhibited creatinine levels higher than 1.1 mg/dl in association with higher levels (*p *< 0.05) of MIF, MCP-1, and IL-6 within the first 24 hours after CPB.

MIF, IL-6, and sCD40L levels were not associated with hematological dysfunction. However, patients with a platelet count lower than 133,000/mm^3 ^exhibited significantly higher levels of MCP-1 at 3, 6, and 10 hours after CPB (Table 2).

Patients who presented minor postoperative neurological dysfunction, especially manifested as behavioral disturbances, had higher levels of MIF and MCP-1 production (*p *< 0.05).

## Discussion

This study suggests that higher perioperative levels of MIF and other inflammatory mediators could be related to specific postoperative organ dysfunction. Importantly, preoperative MIF was directly associated with the intensity of global organ dysfunction measured by MODS on the third day after surgery.

Previous studies have associated sustained high levels of inflammatory markers with poor outcomes in systemic inflammatory response syndrome and sepsis [22]. Present data showed that circulating levels of MIF, IL-6, and MCP-1 increased significantly in response to cardiovascular surgery with CPB, and that maximum levels of MIF occurred before those of IL-6 and MCP-1. Several specific organ dysfunctions had correlations with cytokine production in different times.

First, a significant association between postoperative levels of MIF and pulmonary dysfunction has been reported previously in a study including patients without previous organ dysfunction who were subjected to elective coronary artery bypass surgery [14]. In contrast, the present study also included high-risk patients subjected to more complex cardiovascular surgery in elective and non-elective settings. In this population, postoperative levels of MIF – especially between three and six hours after CPB – were associated not only with pulmonary dysfunction but also with circulatory dysfunction and MODS at the third day after surgery. Experimental models confirmed that MIF is, in fact, implicated in LPS-induced lung injury [22].

Second, postoperative neurological dysfunction was was slightly and rarely associated with postoperative circulating levels of MIF. The present study could not reproduce such an association between cytokine levels and major postoperative neurological complications, possibly because of the small population with postoperative stroke presented in this series. However, minor postoperative neurological complications were associated with higher postoperative levels of MIF and MCP-1.

Third, postoperative levels of MIF and IL-6 were higher in patients presenting postoperative vascular hypo-reactivity, requiring a high dose of vasopressors. There is experimental evidence that MIF is involved with the LPS-induced upregulation of inducible nitric oxide synthase, with its consequent production of nitric oxide and vasodilation [23].

Fourth, MIF can also be involved with LPS-induced myocardial dysfunction, through gene transcription of IL-6 and TNF-α [24]. This study was able to demonstrate an association between higher postoperative levels of MIF and echocardiographically documented ventricular dysfunction (data not shown), but failed to correlate MIF levels with clinical outcomes such as pressure-adjusted heart rate and the need for inotropic agents.

Fifth, as regards postoperative hematological dysfunction, MCP-1 was the only marker to exhibit a consistent relation to postoperative low platelet counts in this study. Previous data indicated that contact with platelets or mesangial cells stimulates the production of MCP-1 through the CD40/CD40L pathway and may contribute to the inflammatory response [25]. In the present study, levels of sCD40L were not directly associated with platelet count but were negatively associated with MCP-1, which could reflect a higher conservation of CD40L on the membrane surface, and therefore would be more liable to intercellular stimulation.

Some questions pertinent to this work could limit its interpretation. The volume of priming solution can be seen as a limitation because of its hemodiluting effect. This procedure could underestimate the early postoperative values of circulating levels of cytokines. Moreover, the lack of determination of the specific sources of cytokines could also interfere with its kinetics, especially considering the cardiopulmonary wash-out at the end of CPB. In spite of these limitations, the main objectives were achieved. Another limitation was the high variability of preoperative levels of sCD40L, which could be attributed to *in vitro *activation of platelets in a population with high-grade atherosclerotic disease; this variation possibly jeopardized the reliable evaluation of perioperative kinetics of this marker and eventual associations between the circulating levels of this substance and postoperative outcomes. Last, the lack of a quantitative analysis of CD40L in cell surfaces would be a better way of clarifying sCD40L kinetics.

It has recently been stated that the pattern of cellular reactivity might be implicated in postoperative organ dysfunction after cardiac surgery in newborns [26]. In addition, the development of postoperative organ edema – and presumably dysfunction – after CBP surgery could be predicted preoperatively, suggesting that it develops in the background of a pre-existing immune activation [27]. One might therefore speculate that the inflammatory milieu, especially reflected by circulating levels of MIF, could render patients susceptibility to an increased inflammatory response [28]. Our results support these findings, suggesting that preoperative immune assessment, focusing especially on circulating levels of MIF, might be associated with postoperative outcome. Despite of the multifactorial nature of specific or multiple organ dysfunctions, MIF shall be explored as a potential therapeutic target in decreasing postoperative complications.

## Conclusion

This study demonstrates that preoperative MIF is consistently associated with multiple organ dysfunction after cardiovascular surgery with CPB. The reproduction of these results in larger and different populations can contribute to the confirmation of its value as a preoperative risk stratification tool.

## Key messages

• 		Cardiopulmonary bypass provokes a systemic spread of inflammatory mediators, which may be related to postoperative organ dysfunction.

• 		Preoperative circulating levels of macrophage migration inhibitory factor (MIF), which is potentially involved in a sustained inflammatory response, may be associated with postoperative organ dysfunction.

• 		The reproduction of these results in a larger population might ratify that MIF is a valuable tool in the prediction of postoperative outcome.

## Abbreviations

CABS = coronary artery bypass surgery; CPB = cardiopulmonary bypass; EuroSCORE = European System for Cardiac Operative Risk Evaluation; FiO_2 _= fraction of inspired oxygen; IL = interleukin; LPS = lipopolysaccharide; MCP = monocyte chemoattractant protein; MIF = macrophage migration inhibitory factor; MODS = multiple organ dysfunction score; PaO_2 _= partial arterial oxygen tension; sCD40L = soluble CD40 ligand; SOFA = Sequential Organ Failure Assessment; TNF = tumor necrosis factor.

## Competing interests

The authors declare that they have no competing interests.

## Authors' contributions

HTFMF was involved in the conception and design of the study and in the analysis and interpretation of data. KCP, MF and DASAV conducted immunoassays and data acquisition and drafted the manuscript. MLFM was involved in patient recruitment and the acquisition of data. LAAC and HCCFN undertook a critical revision of intellectual content. All authors read and approved the final manuscript.

## Figures and Tables

**Table 1:** Demographics and perioperative characteristics of the population under study

Characteristic	Value
Demographic	
Male sex	60 (64.5%)
Age, years	66.2 ± 11.6
Body mass index	25.9 ± 6.5
EuroSCORE	4.58 (2–7)
NYHA I and II, *n*	36 (38.8%)
NYHA III and IV, *n*	57 (61.2%)
Perioperative characteristics (*n *= 93)	
Duration of CPB, minutes)	100 (80–130)
Intraoperative fluid balance, ml/kg per hour	9.09 (6.31–11.36)
Blood transfusion, *n*	45 (48.4%)
CABS, *n*	60 (64.5%)
CABS + open-heart surgery, *n*	14 (15.1%)
Valve replacement/repair, *n*	16 (17.2%)
Aortic surgery, *n*	3 (3.2%)

Errors are SEM; ranges are from first to third quartile. CABS, coronary artery bypass surgery; CPB, cardiopulmonary bypass; EuroSCORE, European System for Cardiac Operative Risk Evaluation; NYHA, New York Heart Association.

**Table 2:** Correlation of time-related cytokine production with organ dysfunctions measured within 24 hours after surgery

Organ dysfunction	Inflammatory markers				*p*
	AI	After CPB			

		3 hours	6 hours	10 hours	

Circulation^a^		MIF			0.017
		IL-10			0.011
				IL-6	0.05
					
Hematological^b^		MCP-1			0.014
			MCP-1		0.026
				MCP-1	0.002
					
Pulmonary^c^	MIF				0.041
		MIF			<0.001
		IL-10			0.032

^a^Defined as noreprinephrine > 1.0 μg/kg per minute; ^b^defined as platelets < 133,000/mm^3^; ^c^defined as a ratio of partial arterial oxygen tension to fraction of inspired oxygen of less than 196. AI, anesthesia induction; CPB, cardiopulmonary bypass; MCP, monocyte chemoattractant protein; MIF, macrophage migration inhibitory factor.

## References

[B1] Butler J, Rocker GM, Westaby S (1993). Inflammatory response to cardiopulmonary bypass. Ann Thorac Surg.

[B2] Andersen LW, Baek L, Degn H, Lehd J, Krasnik M, Rasmussen JP (1987). Presence of circulating endotoxin during cardiac operations. J Thorac Cardiovasc Surg.

[B3] Ruel M, Bianchi C, Khan TA, Liddicoat JR, Voisine P, Araujo E, Lyon H, Kohane IS, Libermann TA, Selke FW (2003). Gene expression after cardiopulmonary bypass and cardioplegic arrest. J Thorac Cardiovasc Surg.

[B4] Wan S, Leclerc JL, Vincent JL (1997). Cytokine responses to cardiopulmonary bypass: lessons learned from cardiac transplantation. Ann Thorac Surg.

[B5] Gessler P, Pretre R, Hohl V, Rousson V, Fischer J, Dahinden C (2004). CXC-chemokine stimulation of neutrophils correlates with plasma levels of myeloperoxidase and lactoferrin and contributes to clinical outcome after pediatric cardiac surgery. Shock.

[B6] Leng L, Metz CN, Fang Y, Xu J, Donnelly S, Baugh J, Delohery T, Chen Y, Mitchell RA, Bucala R (2003). MIF signal transduction initiated by binding to CD74. J Exp Med.

[B7] Leng L, Bucala R (2005). Macrophage migration inhibitory factor. Crit Care Med.

[B8] Bucala R (1996). MIF rediscovered: cytokine, pituitary hormone, and glucocorticoid-induced regulator of the immune response. FASEB J.

[B9] Gando S, Nishihira J, Kobayashi S, Morimoto Y, Nanzaki S, Kemmotsu O (2001). Macrophage migration inhibitory factor is a critical mediator of systemic inflammatory response syndrome. Intensive Care Med.

[B10] Calandra T, Roger T (2003). Macrophage migration inhibitory factor: a regulator of innate immunity. Nat Rev Immunol.

[B11] Beishuizen A, Thijs LG, Haanen C, Vermes I (2001). Macrophage migration inhibitory factor and hypothalamo-pituitary-adrenal function during critical illness. J Clin Endocrinol Metab.

[B12] Das UN (2000). Critical advances in septicemia and septic shock. Crit Care.

[B13] Roger T, David J, Glauser MP, Calandra T (2001). MIF regulates innate immune response through modulation of Toll-like receptor 4. Nature.

[B14] de Mendonça-Filho HTF, Gomes RV, Campos LAA, Tura B, Nunes EM, Gomes R, Bozza F, Bozza PT, Castro-Faria-Neto HC (2004). Circulating levels of macrophage migration inhibitory factor are associated to mild pulmonary dysfunction following cardiopulmonary bypass. Shock.

[B15] Luster AD (1998). Chemokines – chemotactic cytokines that mediate inflammation. N Engl J Med.

[B16] Nannizzi-Alaimo L, Rubenstein MH, Alves VL, Leong GY, Phillips DR, Gold HK (2002). Cardiopulmonary bypass induces release of soluble CD40 ligand. Circulation.

[B17] Wang H, Tracey KJ, Gallin JI, Snyderman R (1999). Tumor necrosis factor, interleukin-6, macrophage migration inhibitory factor and macrophage inflammatory protein-1 in inflammation. Inflammation: Basic Principles and Clinical Correlates.

[B18] Mosmann TR (1994). Properties and functions of interleukin-10. Adv Immunol.

[B19] Nashef SA, Roques F, Michel P, Gauducheau E, Lemeshow S, Salamon R (1999). European system for cardiac operative risk evaluation (EuroSCORE). Eur J Cardiothorac Surg.

[B20] Marshall JC, Cook D, Cristou NV, Sprung CL, Sibbald WJ (1995). Multiple organ dysfunction score: a reliable descriptor of a complex clinical outcome. Crit Care Med.

[B21] Vincent JL, Moreno R, Takala J, Willatts S, De Mendonca A, Bruining H, Reinhart CK, Suter PM, Thijs LG (1996). The SOFA (Sequential Organ Failure Assessment) score to describe organ dysfunction/failure. On behalf of the Working Group on Sepsis-Related Problems of the European Society of Intensive Care Medicine. Intensive Care Med.

[B22] Bozza M, Satoskar AR, Lin G, Lu B, Humbles AA, Gerard C, David JR (1999). Targeted disruption of migration inhibitory factor gene revels its critical role in sepsis. J Exp Med.

[B23] Huang XR, Chunhui CW, Chen YX (2001). Macrophage migration inhibitory factor is an important mediator in the pathogenesis of gastric inflammation in rats. Gastroenterology.

[B24] Chanon F, Metz CN, Bucala R, Lestur O (2005). Endotoxin-induced myocardial dysfunction: effects of macrophage migration inhibitory factor neutralization. Circ Res.

[B25] Tanaka T, Kuroiwa T, Ikeuchi H, Ota F, Kaneko Y, Ueki K, Tsukada Y, Mcinnes IB, Boumpas DT, Nojima Y (2002). Human platelets stimulate mesangial cells to produce monocyte chemoattractant protein-1 via the CD40/CD40 ligand pathway and may amplify glomerular injury. J Am Soc Nephrol.

[B26] Schumacher K, Korr S, Vazques-Jimenez JF, von Bernuth G, Duchateau J, Seghaye M (2005). Does cardiac surgery in newborn infants compromise blood cell reactivity to endotoxin?. Crit Care.

[B27] Bocsi J, Hambsch J, Osmancik P, Schneider P, Valet G, Tárnok A (2002). Preoperative prediction of pediatric patients with effusions and edema following cardiopulmonary bypass surgery by serological and routine laboratory data. Crit Care.

[B28] Leng L, Bucala R (2005). Macrophage migration inhibitory factor. Crit Care Med.

